# Low-rank tensor methods for Markov chains with applications to tumor progression models

**DOI:** 10.1007/s00285-022-01846-9

**Published:** 2022-12-02

**Authors:** Peter Georg, Lars Grasedyck, Maren Klever, Rudolf Schill, Rainer Spang, Tilo Wettig

**Affiliations:** 1 Department of Physics, University of Regensburg, 93040 Regensburg, Germany; 2 Institute for Geometry and Applied Mathematics, RWTH Aachen University, 52062 Aachen, Germany; 3 Department of Statistical Bioinformatics, Institute of Functional Genomics, University of Regensburg, 93040 Regensburg, Germany

**Keywords:** Transient distribution, Stochastic Automata Networks, Mutual Hazard Networks, Hierarchical Tucker format, 15A69, 60J22, 60J28

## Abstract

Cancer progression can be described by continuous-time Markov chains whose state space grows exponentially in the number of somatic mutations. The age of a tumor at diagnosis is typically unknown. Therefore, the quantity of interest is the time-marginal distribution over all possible genotypes of tumors, defined as the transient distribution integrated over an exponentially distributed observation time. It can be obtained as the solution of a large linear system. However, the sheer size of this system renders classical solvers infeasible. We consider Markov chains whose transition rates are separable functions, allowing for an efficient low-rank tensor representation of the linear system’s operator. Thus we can reduce the computational complexity from exponential to linear. We derive a convergent iterative method using low-rank formats whose result satisfies the normalization constraint of a distribution. We also perform numerical experiments illustrating that the marginal distribution is well approximated with low rank.

## Introduction

The dynamics of cancer can be studied using tumor progression models, cf. (Beerenwinkel et al. 2014). These models describe the evolving genotype of a tumor as a continuous-time Markov chain. A model includes *d* genomic loci that may or may not be mutated. It starts out with all mutations absent and then progressively accumulates mutations (or other genomic events such as copy number alterations). The number of possible states of the tumor is thus $2^d$. In typical applications one is interested in probability distributions over this state space that are far from stationary. Extrapolating the future course of a given tumor requires the computation of transient distributions. However, since the age of a tumor and thus the time point of an observation of the Markov chain is generally unknown, we study the transient distribution integrated over an exponentially distributed observation time, called the *time-marginal distribution*. It can be obtained as the solution of a large linear system. Today at least $d = 299$ genes are known to drive tumor progression, cf. (Bailey et al. 2018), i.e., a single distribution in $\mathbb {R}^{2^{299}}$ would require the storage of more entries than there are atoms in the observable universe. This phenomenon is called *state-space explosion* (Buchholz and Dayar 2007) and renders classical methods for calculating or storing distributions infeasible. This problem is also known in other application areas, e.g., chemical-reaction networks (Anderson et al. 2010), the chemical master equation (Kazeev et al. 2014), Hamiltonian dynamics (Haegeman et al. 2016), queuing networks (Chan 1987), evolutionary dynamics (Niederbrucker and Gansterer 2011) or stochastic neural networks (Yamanaka et al. 1997).

Our main goal is to develop a method for calculating or approximating the marginal distribution. Due to the state-space explosion, methods for calculating the entire marginal distribution in the context of tumor progression have been limited to about $d = 25$ genomic events (Schill et al. 2019). In (Gotovos et al. 2021) it is demonstrated that learning models of this size from data is an underspecified problem and that increasing the number *d* can make inference more robust. This requires a tractable approximation of the marginal distribution. To allow for a probabilistic interpretation of this approximation, the latter should be a probability distribution itself, i.e., all entries should be non-negative, and the sum of all entries should be equal to one. Here, we neglect the non-negativity to ensure an error-controlled approach, see, e.g., (Kim et al. 2013) for an overview. So the following questions need to be answered:

1. How can we overcome the state-space explosion?
2. Subsequently, how can we determine marginal distributions?
3. At the same time, how can we ensure that a solution sums to one?

In order to address question 1 we use so-called *low-rank tensor formats*. These are known to reduce the cost from exponential to linear in the number of events *d* provided the distribution exhibits a low-rank structure.

To do so, we model Markov chains of interacting processes as *Stochastic Automata Networks* (Plateau and Stewart 2000). These allow for a representation of the infinitesimal generator as a sum of Kronecker products. In the context of low-rank tensors, such a representation is referred to as *CANDECOMP/PARAFAC (CP) format* (Carroll and Chang 1970; Harshman 1970), and the number of elements in this sum is called *CP rank*. As there are dependencies between individual processes, the transition rates, i.e., entries of the generator, depend on the current state of the Markov chain. We model the transition rates in a separable way where each factor is defined by the current state in one automaton.

Based on the CP representation of the infinitesimal generator, such representations can also be derived for the operator as well as for the right-hand side of the linear system defining the marginal distribution. We use these low-rank tensor representations to overcome the state-space explosion, cf. question 1, and to compute the marginal distribution, cf. question 2

Strategies which have been used so far to calculate the marginal distribution cannot be generalized to low-rank tensors. Existing methods for solving general systems in low-rank tensor formats can be roughly divided into optimization-based approaches and iterative procedures, see, e.g., (Grasedyck et al. 2013) for an overview. Here we focus on iterative ones and discuss related works in Sect. 3.2. In low-rank formats, the effort for storage and execution of arithmetic operations depends, as the name already suggests, on the rank. Arithmetic operations needed in iterative procedures lead to an increase in the representation rank. One way to counteract this rank increase is the so-called *truncation*, i.e., the approximation of a tensor by one of lower rank. This allows for reducing storage and computational costs and thereby for efficient iterative procedures in low-rank formats. Beside the CP format there are several other low-rank tensor formats known. Here we focus in particular on the *hierarchical Tucker format* (Hackbusch and Kühn 2009; Grasedyck 2010). A special feature of these compared to the CP format is the possibility of accurate truncation. In (Hackbusch et al. 2008) it is proven that a convergent iterative method which is supplemented by truncation still converges if the approximation error is small enough.

While guaranteeing convergence, iterative methods supplemented by truncation do not ensure that their results sum to one anymore. In order to address question 3, we derive a novel iterative method based on the Neumann series (Dubois et al. 1979) and the uniformization method (Grassmann 1977) using low-rank tensor formats. We verify that its result sums up to one and prove its convergence. In our numerical experiments, we focus on the concept of *Mutual Hazard Networks* (Schill et al. 2019) for tumor progression. Our experiments illustrate that the marginal distribution can be approximated by low-rank tensors using our new algorithm.

This work is organized as follows. In Sect. 2 we derive the linear system that defines the time-marginal distribution of a continuous-time Markov chain. Starting from the concept of Mutual Hazard Networks and Stochastic Automata Networks explained there, we define a model class of Markov chains describing interacting processes. For these we will then derive low-rank tensor representations for the operator and the right-hand side of the linear system. In Sect. 3 we introduce the concept of tensors. We review the hierarchical Tucker format and discuss related work. Using these formats we derive an iterative method and prove its convergence. In Sect. 4 we perform numerical experiments based on the concept of Mutual Hazard Networks. In Sect. 5 we conclude.

## Statement of the problem and modeling

### Time-marginal distribution

A continuous-time Markov chain is defined by its state space $S$, its infinitesimal generator $\mathsf {Q}$ and an initial distribution $\mathsf {q}$. In this paper we assume that the state space $S$ is discrete. The generator is an operator $\mathsf {Q}\in \mathbb {R}^{S\times S}$, i.e., a linear mapping from $\mathbb {R}^{S}$ to $\mathbb {R}^{S}$, which stores the rates of transition from state *x* to another state *y* in $\mathsf {Q}_{y,x} \ge 0$ and the rates of staying in a state *x* in $\mathsf {Q}_{x,x} \le 0$. By construction each column of $\mathsf {Q}$ sums up to 0.^1^ The probability distribution $\mathsf {p}(t)$ as function of the time $t\ge 0$ is defined as the solution of the initial value problem

1 $$\begin{aligned} {\left\{ \begin{array}{ll} \frac{\mathrm {d}}{\mathrm {d} t}\mathsf {p}(t) &{}= \mathsf {Q}\mathsf {p}(t) \qquad \text { for all } t \ge 0,\\ \quad \mathsf {p}(0) &{}= \mathsf {q}, \end{array}\right. } \end{aligned}$$

i.e., $\mathsf {p}(t) = \exp \left( \mathsf {Q}t \right) \mathsf {q}$ for all $t \ge 0$. We make the common assumption that every trajectory starts at the same state, i.e., the initial distribution $\mathsf {p}(0)= \mathsf {q}\in \mathbb {R}^{S}$ is a canonical unit vector.

Here, we assume that the observation time *t* is unavailable, such as, e.g., in tumor progression modeling, and thus must be treated as a random variable. Therefore, we are interested in a so-called *time-marginal distribution* $\mathsf {p}$ which is independent of the time *t* and which we will call marginal distribution for brevity. Each entry $\mathsf {p}_x$ of the marginal distribution indicates the probability of observing a state $x \in S$ at a random time. We follow the common assumption that the sampling time is an exponentially distributed random variable with rate 1, i.e., $t \sim {\text {Exp}}[1]$. Similar approaches can be found, e.g., in Hjelm et al. (2006); Beerenwinkel and Sullivant (2009); Schill et al. (2019); Gotovos et al. (2021). Please note that for general ${\tilde{t}} \sim {\text {Exp}}[\lambda ]$ with $\lambda > 0$ the same results can be obtained by simply rescaling the time, i.e., $t:= \frac{{\tilde{t}}}{\lambda } \sim {\text {Exp}}[1]$. Here, we have

2 $$\begin{aligned} \mathsf {p}:= \int \limits _{0}^{\infty } \exp (-t) \mathsf {p}(t)~ \mathrm {d} t = \int \limits _{0}^{\infty } \exp (t \left( \mathsf {Q}- {\mathsf {Id}}\right) ) \mathsf {p}(0)~ \mathrm {d} t . \end{aligned}$$

The following lemma shows that this integral exists.

#### Lemma 1

Let $\mathsf {Q}\in \mathbb {R}^{S\times S}$ be the infinitesimal generator of a continuous-time Markov chain over the discrete state space $S$. Then the integral (2) exists and

3 $$\begin{aligned} \mathsf {p}= \left( {\mathsf {Id}}- \mathsf {Q}\right) ^{-1} \mathsf {p}(0). \end{aligned}$$

#### Proof

For the existence of the improper integral we show that the spectrum $\sigma \left( \mathsf {Q}- {\mathsf {Id}}\right)$ of the operator $\mathsf {Q}- {\mathsf {Id}}$ fulfills $\sigma \left( \mathsf {Q}- {\mathsf {Id}}\right) \subseteq \mathbb {C}^{-}:= \{z \in \mathbb {C}~\vert ~ {\text {Re}}(z) < 0 \}$. Applying the Gershgorin circle theorem (Gershgorin 1931) for $\mathsf {Q}$ with $\mathsf {Q}_{x,x} = - \sum \limits _{y \ne x} \mathsf {Q}_{y,x} \le 0$, we obtain

$$\begin{aligned} \sigma (\mathsf {Q}) \subseteq \bigcup \limits _{x \in S} \big \{ z \in \mathbb {C}~\big \vert ~ \big \vert z- \mathsf {Q}_{x,x} \big \vert \le \big \vert \mathsf {Q}_{x,x} \big \vert \big \} \subseteq \big \{z \in \mathbb {C}~ \big \vert ~ {\text {Re}}(z) \le 0 \big \} \end{aligned}$$

and thus, $\sigma \left( \mathsf {Q}- {\mathsf {Id}}\right) \subseteq \{z \in \mathbb {C}~ \vert ~ {\text {Re}}(z) \le -1 \}$. Then the statement follows from direct calculation.

This result can also be obtained by understanding the process as an absorbing Markov chain where from each state a transition with rate 1 enters the absorbing state. Then the time-marginal distribution equals the distribution just before absorption, see also (Gotovos et al. 2021, Proposition 2). From this point of view, the statement follows from the theory of absorbing Markov chains. $\square$

Hence, the marginal distribution $\mathsf {p}$ is defined as the unique solution of a linear system, since the operator ${\mathsf {Id}}- \mathsf {Q}$ is regular.

Next, we specify the class of Markov chains for which we compute the time-marginal distribution $\mathsf {p}$. We start with an introduction into a specific type of model used in tumor progression and then generalize this type based on the concept of *Stochastic Automata Networks* (Plateau and Stewart 2000). All these Markov chains will offer a sparse representation of the infinitesimal generator $\mathsf {Q}$ in order to overcome the state-space explosion.

### Modeling tumor progression via Mutual Hazard Networks

As mentioned in the introduction, tumor progression can be modeled as continuous-time Markov chain over a discrete state space $S$. Typically, one is interested in a transient distribution, but the time point of observation, i.e., the age of the tumor, is unknown. Nevertheless, in order to be able to make statements about the probability distribution for all possible tumors, the time-marginal distribution is needed.

For tumor progression, the state space $S$ can be modeled as follows. By consideration of *d* genomic events, as point mutations, copy number alterations, or changes in DNA methylation, each state $x \in S$ represents the genotype of a tumor by indicating whether a genomic event has occurred or not. Modelling a state $x = (x_1, \dots , x_d) \in S$ as a vector of length *d* with $x_i = 1$, if event *i* has occurred, and otherwise $x_i = 0$, the set of all possible states (or tumors respectively) can be represented as4Thus, the number of possible tumors increases exponentially in the number *d* of genomic events considered, also known as state-space explosion.

In tumor progression modeling, the transition rates are usually unknown, and therefore certain assumptions have to be made. Here we focus on the concept of *Mutual Hazard Networks* (Schill et al. 2019) which offers a sparse representation of the infinitesimal generator $\mathsf {Q}$. In (Schill et al. 2019) the following assumptions define a Mutual Hazard Network:

(i) All events are assumed to occur one after another.
(ii) All events are irreversible, i.e., there are no transitions from state $x \in S$ with $x_{i} = 1$ to $y \in S$ with $y_{i} = 0$ for an event *i*.
(iii) The occurrence of an event depends on the genotype of the tumor in a separable way. This means that there are *mutual* effects between events on their rate of occurrence which can be factorized, and each factor is described by a certain event.
(iv) Genomic events that have not occurred yet have no effect on the transition rates of all others.

According to assumption (iii) a first-order Cox proportional hazard model (Cox 1972) is used to specify the transition rates, i.e., there are parameters $\mathsf {\Theta }\in \mathbb {R}^{d \times d}$ defining the mutual effects on transition rates from state *x* to *y*,

5 $$\begin{aligned} \mathsf {Q}_{y, x} = \mathsf {\Theta }_{i,i} \prod \limits _{\begin{array}{c} j \in \{1, \dots , d\},\\ x_j = 1 \end{array}}\mathsf {\Theta }_{i,j}, \end{aligned}$$

where *x* and *y* only differ in one event *i* with $x_i = 0 < y_i = 1$. To be precise, the parameter $\mathsf {\Theta }_{i,j} := \mathsf {\Theta }(x_i \rightarrow y_i, x_j)\ge 0$ is the (multiplicative) effect of state $x_j$ for event *j* on the transition from $x_i$ to $y_i$ for event *i* for events $i \ne j$. The parameter $\mathsf {\Theta }_{i,i} := \mathsf {\Theta }(x_i \rightarrow y_i, x_i) \ge 0$ is then the baseline rate of transition from $x_i$ to $y_i$ for event *i*. Following (iv) all effects $\mathsf {\Theta }(x_i \rightarrow y_i, x_j)$ with $x_j = 0$ and $i \ne j$ are equal to 1, i.e., they are neutral multiplicative effects in (5).

**Fig. 1:**
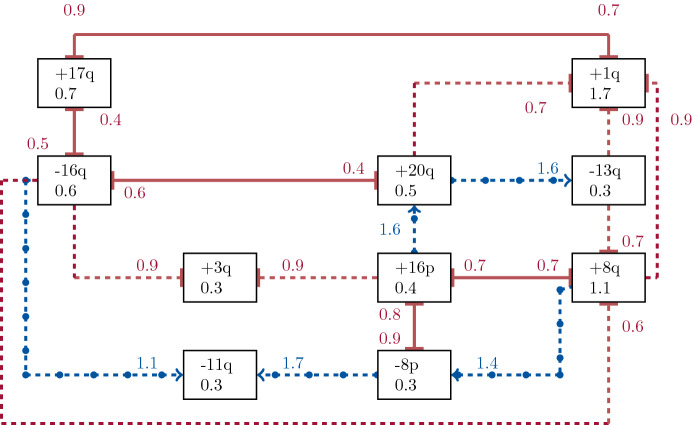
Mutual Hazard Network for breast cancer, see (Schill et al. 2019, supplementary material Figure 1): Each box represents a genomic event and each line a direct effect between them. A dashed line identifies a unilateral effect, a solid reciprocal effects, a red line (without marks) an inhibiting and a blue one (with circles) a promoting effect

This modeling allows for describing different mutual effects between genomic events on their transition rates, see (iii). Figure 1 (Schill et al. 2019, supplementary material Figure 1) shows the Mutual Hazard Network inferred for breast cancer data.

Each box represents a genomic event relevant to breast cancer and each line a direct effect between them. A dashed line shows a unilateral effect and a solid line identifies a reciprocal effect. In addition, we can distinguish effects based on their nature and magnitude. In the present case, an amplification on the p-arm of the 16th chromosome (event +16p) inhibits a deletion on the p-arm of the 8th chromosome (event -8p) with $\mathsf {\Theta }_{-8p, +16p} = 0.9$. Such an inhibitory effect of event *j* on *i* is indicated in the network by a red connection (lines without marks) and corresponds to a parameter $\mathsf {\Theta }_{i,j} < 1$. Similarly, event +16p promotes an amplification on the q-arm of the 20th chromosome (event +20q) with $\mathsf {\Theta }_{+20q, +16p} = 1.6$. A promoting effect of event *j* on *i* is indicated by blue connection (lines with circles) in the network and corresponds to a parameter $\mathsf {\Theta }_{i,j} > 1$. Neutral effects are represented in Fig. 1 by the absence of edges, e.g., event +16p is not connected to event +1q which means that event +16p does not affect event +1q directly. At the level of parameters this corresponds to a multiplicative effect of $\mathsf {\Theta }_{i,j} = 1$. As can already be seen in Fig. 1, in typical applications most of the direct effects between events are assumed to be neutral. Beyond these direct effects, events can also influence each other indirectly. In the example shown, event +16p is not associated with event +1p, so it has a neutral direct effect on event +1p. However, event +16p favors the occurrence of event +20q, which itself inhibits event +1p. Excluding all other effects, event +16p could therefore indirectly have an inhibitory effect on event +1p (despite $\mathsf {\Theta }_{+1q, +16p} = 1$).

The diagonal entries of $\mathsf {Q}$ follow directly from (5) since each column sums up to 0, i.e., $\mathsf {Q}_{x,x} = - \sum _{y \ne x} \mathsf {Q}_{y, x}$. Together with the separation in (5) this allows for the following representation of the infinitesimal generator, cf. (Schill et al. 2019):

6 $$\begin{aligned} \mathsf {Q}= \sum \limits _{i = 1}^d \bigotimes \limits _{j < i} \begin{pmatrix} 1 &{} 0 \\ 0 &{}\mathsf {\Theta }_{i, j} \end{pmatrix} \otimes \begin{pmatrix} -\mathsf {\Theta }_{i,i} &{} 0 \\ \mathsf {\Theta }_{i,i} &{} 0 \end{pmatrix} \otimes \bigotimes \limits _{j > i} \begin{pmatrix} 1 &{} 0 \\ 0 &{}\mathsf {\Theta }_{i, j} \end{pmatrix}. \end{aligned}$$

Instead of $\vert S\vert \cdot \vert S\vert = 2^{2d}$ entries, one only has to store $4 d^2$ entries. In the following, we will observe that the structure in (6) of the operator $\mathsf {Q}$ will allow us to use tensor formats for the calculation of the time-marginal distribution.

While this is a specific model for tumor progression on genomic data, the question arises whether a similar structure of $\mathsf {Q}$ can be used for more general models with a larger range of applications. In general, the progression of disease is not an irreversible process, and symptoms and traits can arise and then abate, such as inflammation (Zhao et al. 2021). They also do not necessarily have to be modeled in a binary way, but can have different levels of severity, such as fever (Johnston et al. 2019). The same formalism can be used to model the attrition and maintenance of technical systems of interacting components (Amoia et al. 1981). To do this, we introduce the concept of *Stochastic Automata Networks* (Plateau and Stewart 2000) which are known to offer a similar representation for the infinitesimal generator $\mathsf {Q}$. We note that the Markov chains defined by a Mutual Hazard Network also belong to this class of Stochastic Automata Networks.

### Stochastic automata networks

For a continuous-time Markov chain of interacting processes the discrete state space factorizes in a natural way into the state spaces of the individual processes. Each process is itself a Markov chain over its own state space $S_{i}$ and is called a *stochastic automaton* $\mathcal {A}_{i}$. The set $\{\mathcal {A}_1, \dots , \mathcal {A}_d\}$ of *d* stochastic automata is called a *Stochastic Automata Network*, cf. (Plateau and Stewart 2000). The full state space $S$ consists of all possible combinations of states in each automaton, i.e., for $n_{i} := \vert S_{i} \vert$ and $n := \max _{i} n_{i}$ it is given by7Each state $x = (x_1, \dots , x_d) \in S$ specifies its state $x_i \in S_{i}$ in each automaton $\mathcal {A}_{i}$. Already at this point we note overlaps with the concept of Mutual Hazard Networks. Each genomic event *i* of the Mutual Hazard Network represents a stochastic automaton $\mathcal {A}_i$ with local state space $S_i = \{0, 1\}$. This results in the global state space $S$ for *d* genomic events in (4).

We now consider transitions between states in the full state space $S$, which are given by one or more transitions between states within the individual state spaces $S_{i}$.^2^ It is known that the infinitesimal generator $\mathsf {Q}$ of a Stochastic Automata Network can be represented as a sum of Kronecker products, i.e., for *d* automata and *m* transitions it is given by

8 $$\begin{aligned} \mathsf {Q}= \sum \limits _{i = 1}^{2 m + d} ~ \bigotimes \limits _{j = 1}^d \mathsf {Q}_{i}^{(j)} \quad \text { with } \mathsf {Q}_{i}^{(j)} \in \mathbb {R}^{S_i \times S_i}, \end{aligned}$$

cf. (Plateau and Stewart 2000). Again, instead of $\vert S\vert \cdot \vert S\vert \le n^{2d}$ entries, one only has to store $\left( 2 m + d\right) d n^2$ entries.

A specific way to define the matrices $\mathsf {Q}_{i}^{(j)}$ is given in (6) for a Mutual Hazard Network, where the number of possible transitions *m* is equal to the number of events *d*. Note that the reduction of $2m+d$ to *d* terms in the sum results from the separability of the transition rates in (5).

Based on the formalism of Stochastic Automata Networks we extend the concept of Mutual Hazard Networks. On the one hand, the separability and parameterization should be preserved. On the other hand, an extension of the models and an enlargement of the possible application areas should be made possible by relaxing the assumptions.

### Generalized modeling

We focus on a continuous-time Markov chain represented as a Stochastic Automata Network with *d* automata $\{\mathcal {A}_1, \dots , \mathcal {A}_d\}$. As in Sect. 2.3 each automaton $\mathcal {A}_{i}$ has a state space $S_{i}$ with $\vert S_{i} \vert = n_{i}$, and the state space of the network is given by . According to (i) and (iii) we make the following assumptions:

(I) There is only one transition in one automaton at a time.
(II) Each transition depends on the current global state in a separable way, i.e., each transition rate can be factorized, and each factor is described by the current local state in one automaton.

For each automaton $\mathcal {A}_i$ we denote the set of transitions in $\mathcal {A}_{i}$ by $T_{i} \subseteq \{ x_i \rightarrow y_i ~ \vert ~ x_i \ne y_i \in S_i \}$. Again, we model the mutual effects on local transitions using parameters $\mathsf {\Theta }$. Each parameter $\mathsf {\Theta }( t_i, x_j) \ge 0$ is the effect of state $x_j$ in automaton $\mathcal {A}_j$ on the transition $t_i \in T_i$ in automaton $\mathcal {A}_i$ for $i \ne j$ and the baseline rate for $i= j$. Thus, the transition rate from state *x* to *y* in $S$, where *x* and *y* differ only in automaton $\mathcal {A}_{i}$ and satisfy $t_i = (x_i \rightarrow y_i) \in T_i$, can be represented with parameters $\mathsf {\Theta }( t_i, x_j)$ by

$$\begin{aligned} \mathsf {Q}_{y, x} = \prod \limits _{j = 1}^d \mathsf {\Theta }( t_i, x_j). \end{aligned}$$

The diagonal entries of the generator are again given by $\mathsf {Q}_{x,x} = - \sum _y \mathsf {Q}_{y, x}$, and thus we specify a data-sparse representation by

9 $$\begin{aligned} \mathsf {Q}= \sum \limits _{i=1}^d \sum \limits _{ t_i \in T_i} \bigotimes \limits _{j =1}^d \mathsf {Q}^{(t_i)}_j \quad \text { with } \mathsf {Q}^{(t_i)}_j \in \mathbb {R}^{S_j \times S_j}. \end{aligned}$$

For $i \ne j$, $\mathsf {Q}^{(t_i)}_j$ is a diagonal matrix containing the effects of all states in automaton $\mathcal {A}_j$ on the transition $t_i$ in $\mathcal {A}_i$, i.e., its diagonal entries are given by the parameters $\mathsf {\Theta }(t_i, x_j)$ for all states $x_j \in S_j$ in automaton $\mathcal {A}_j$. For $i=j$, the matrix $\mathsf {Q}^{(t_i)}_i$ contains only two non-zero entries: The entry corresponding to $t_i = (x_i \rightarrow y_i)$ is the baseline rate $\mathsf {\Theta }(t_i, x_i)$, and the diagonal entry at $x_i$ is $-\mathsf {\Theta }(t_i, x_i)$. Similar to the representation of $\mathsf {Q}$ for the Mutual Hazard Networks in (6), the number of terms in the sum is given by the overall number of possible transitions in the Markov chain, i.e., $\sum _{i=1}^d \vert T_i \vert$. Compared to the general representation (8) for Stochastic Automata Networks, the reduction of terms is possible due to the separability of transition rates (II).

In contrast to the model based on Mutual Hazard Networks, this generalized version allows for different and larger local state spaces (instead of binary $S_i = \{0, 1\}$ for all *i*) and does not require specific restrictions as irreversibility or specific neutral effects of states. In particular, the automata may be cyclic. While the data-sparse structure of the infinitesimal generator $\mathsf {Q}$ based on certain parameters $\mathsf {\Theta }$ is preserved. In the numerical experiments, we will then refer to the case of Mutual Hazard Networks, which have already been used in practical simulations for tumor progression (Schill et al. 2019; Gotovos et al. 2021).

### Structure of the linear system

As mentioned in Sect. 1, the solution of the linear system

10 $$\begin{aligned} \left( {\mathsf {Id}}- \mathsf {Q}\right) \mathsf {p}= \mathsf {p}(0)\end{aligned}$$

in Schill et al. (2019) based on classical methods was limited to about $d < 25$ automata. In order to overcome the state-space explosion, we need to go beyond classical methods. We have already given a data-sparse representation of $\mathsf {Q}$, also in the more general case of Sect. 2.4. Note that the identity ${\mathsf {Id}}= \bigotimes _{i=1}^d {\mathsf {Id}}_{S_i}$ and the initial distribution $\mathsf {p}(0)= \mathbf {e}_z = \bigotimes _{i = 1}^d \mathbf {e}_{z_i}$ with initial state $z \in S$ can be written as Kronecker products, where ${\mathsf {Id}}_{S_i} \in \mathbb {R}^{S_i \times S_i}$ is the identity operator on $\mathbb {R}^{S_i}$ and $\mathbf {e}_{z_i} \in \mathbb {R}^{S_i}$ is the $z_i$-th canonical unit vector. Hence, the operator and the right-hand side of (10) have a representation as a short sum of *d* Kronecker products, which allows for efficient storage. We still need a low-rank method to solve the linear system, which is the subject of the next section.

## Low-rank method to compute the marginal distribution

We now compute the marginal distribution $\mathsf {p}$ in a data-sparse way. To avoid losing this sparsity when performing arithmetic operations we regard our operators and distributions as *tensors* by interpreting the Kronecker products as tensor products.

### Low-rank tensor formats

We view tensors as multidimensional generalizations of vectors and matrices, i.e., of one-dimensional and two-dimensional tensors.

#### Definition 1

(tensor) Let $d \in \mathbb {N}$ and $\mathcal {I} = \times _{i=1}^d \mathcal {I}_{i}$ be a Cartesian product of discrete index sets $\mathcal {I}_{i}$. An object $\mathsf {B}\in \mathbb {R}^{\mathcal {I}}$ is called a *tensor* of *dimension* *d*. Each direction $i \in \{1, \dots , d\}$ is called a *mode* of $\mathsf {B}$, and the cardinality of the *i*-th index set $\vert \mathcal {I}_{i} \vert$ is called the *i*-*mode size*.

In our case, the index set $\mathcal {I}$ corresponds to the state space , our distributions $\mathsf {p}$, $\mathsf {p}(0)\in \mathbb {R}^S$ are tensors of dimension *d*, and the automata $\mathcal {A}_{i}$ correspond to the modes with sizes $n_{i} = \vert S_{i} \vert$.

In the language of tensors, the structure of $\mathsf {Q}$ in (9) is an example of the so-called *CANDECOMP/PARAFAC (CP) format* introduced in Carroll and Chang (1970); Harshman (1970).

#### Definition 2

(CP format) A tensor $\mathsf {B}\in \mathbb {R}^{\mathcal {I}}$ has a *CP representation* if there exist $\mathbf {b}_{i}^{\left( j\right) } \in \mathbb {R}^{\mathcal {I}_{j}}$ such that

11 $$\begin{aligned} \mathsf {B}= \sum \limits _{i = 1}^r \bigotimes \limits _{j = 1}^d \mathbf {b}_{i}^{\left( j \right) }. \end{aligned}$$

Then $r \in \mathbb {N}_0$ is called the *CP representation rank*, and the $\mathbf {b}_{i}^{\left( j\right) }$ are called the *CP factors* of $\mathsf {B}$. The minimal $r \in \mathbb {N}_0$ such that $\mathsf {B}$ has such a CP representation (11) is called the *CP rank* of $\mathsf {B}$.

The infinitesimal generator $\mathsf {Q}$ in (9) has CP representation rank $\sum _{i = 1}^d \vert T_{i}\vert \le d n^2$. The identity ${\mathsf {Id}}$ as well as the right-hand side $\mathsf {p}(0)$ have CP rank 1. A core advantage of the CP format is the data sparsity in case of small representation rank *r*: The representation (11) of a tensor $\mathsf {B}\in \mathbb {R}^{\mathcal {I}}$ has storage complexity in $\mathcal {O}\bigl ( r \sum _{i = 1}^d n_{i} \bigr ) = \mathcal {O}(rdn)$ in contrast to $\mathcal {O}\bigl (\prod _{i = 1}^d n_{i}\bigr ) = \mathcal {O}\left( n^d\right)$ for the full tensor. We use the $\mathcal {O}$-notation for storage and computational costs to describe that costs in $\mathcal {O}(f(\omega ))$ grow asymptotically no faster than $\text {const} \cdot f(\omega )$ for a constant $\text {const} > 0$ and a function *f* depending on certain value $\omega$.

Since we want to compute the marginal distribution $\mathsf {p}$, we have to solve a linear system whose operator and right-hand side have a CP representation each. For an operator with CP rank $r > 1$ it is unknown how to calculate its inverse analytically. Hence, we need a solver and arithmetic operations to compute the solution numerically. Performing arithmetic operations such as adding two tensors and applying an operator results in an increase in the representation rank. In the CP format, this increase in representation ranks can be traced easily: For example, if we add two tensors $\mathsf {B}_1$ and $\mathsf {B}_2$ with representation ranks $r_1$ and $r_2$ by appending the CP factors of $\mathsf {B}_2$ to those of $\mathsf {B}_1$, the sum $\mathsf {B}= \mathsf {B}_1 + \mathsf {B}_2$ already has representation rank $r_1 + r_2$. Similarly, applying a CP operator with representation rank $r_1$ to a CP tensor with representation rank $r_2$, by applying the operator factor by factor to the CP tensor, results in a CP tensor with representation rank $r_1 \cdot r_2$.

To counteract this increase in the representation rank, arithmetics in low-rank formats is supplemented by a so-called *truncation*, i.e., approximating a tensor with one of lower representation rank. As the set of tensors with CP rank at least *r* is not closed for $d > 2$, low-rank approximation within the CP format is an ill-posed problem, see de Silva and Lim (2008), and usually optimization-based methods are used for this purpose. However, we overcome this drawback by using tensor formats that allow for truncation based on the singular value decomposition of matrices.

To do so, a high-dimensional tensor is reshaped into a matrix by selecting modes defining its rows, while all others define its columns. The resulting matrix, called *matricization*, is defined, following (Grasedyck 2010), as follows.

#### Definition 3

(matricization) Let $\mathsf {B}\in \mathbb {R}^{\mathcal {I}}$ and $t \subseteq \{1, \ldots , d\}$ with $t \ne \emptyset$ and $s = \{1, \ldots , d\}\setminus t$. The *matricization* of $\mathsf {B}$ corresponding to *t* is defined as $\mathsf {B}^{(t)} \in \mathbb {R}^{\mathcal {I}_t \times \mathcal {I}_s}$ with $\mathcal {I}_t = \prod \limits _{i \in t} \mathcal {I}_{i}$ and

$$\begin{aligned} \mathsf {B}^{(t)}_{(x_{i})_{{i} \in t},(x_{i})_{{i} \in s}}= \mathsf {B}_{x_1,\dots ,x_d} \end{aligned}$$

for all $x=(x_{i})_{{i} \in \{1, \ldots , d\}} \in \mathcal {I}$. In particular $\mathsf {B}^{(\{1, \ldots , d\})} \in \mathbb {R}^{\mathcal {I}}$.

**Fig. 2:**
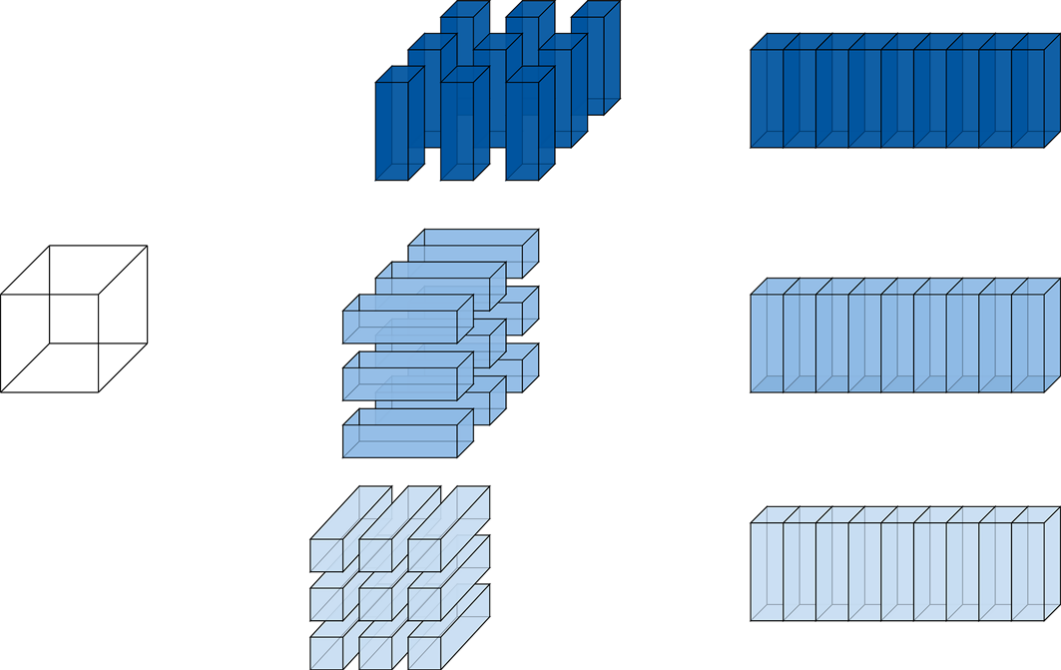
Matricizations of a three-dimensional tensor corresponding to the single modes $t = \{1\}, \{2\}$ and $\{3\}$

The matricizations of a three-dimensional tensor corresponding to the single modes $\{1\}, \{2\}$ and $\{3\}$ are visualized in Fig. 2.

For a matricization of a tensor, the classical singular value decomposition can be used for low-rank approximation. So-called *tree tensor formats* make use of this observation. A popular one is the *tensor train format* which was first introduced to the numerical analysis community in Oseledets and Tyrtyshnikov (2009). It is also known in other areas as *matrix product states* (White 1992; Östlund and Rommer 1995) or as *linear tensor network* (van Loan 2008).

Here we focus on the *hierarchical Tucker format* which was first introduced in Hackbusch and Kühn (2009) and further analyzed in Grasedyck (2010). As the name suggests, the hierarchical Tucker format is based on a hierarchical subdivision of the modes $\{1, \ldots , d\}$. This bisection of the modes is described by a binary *dimension tree*. The set of all modes is distributed from the root to the children until only single element subsets are left in the leaves. Formally, a dimension tree can be defined as follows:

#### Definition 4

(dimension tree) A *dimension tree* $\mathcal {T}$ for dimension $d \in \mathbb {N}$ is a binary tree with nodes labeled by non-empty subsets of $D:= \{1, \dots , d\}$. Its root is labeled with *D* and each node *t* (identified with its label) satisfies one and only one of the following conditions:

1. $t \in \mathcal {L}({\mathcal {T}})$ is a leaf of $\mathcal {T}$ and labeled by a single element subset $t =\{i\} \subseteq D$.
2. $t \in \mathcal {I}({\mathcal {T}}):= \mathcal {T}\setminus \mathcal {L}({\mathcal {T}})$ is an inner node of $\mathcal {T}$ and has exactly two children $t_1, t_2$ which fulfill $t = t_1 \cup t_2$ and $t_1 \cap t_2 = \emptyset$.

**Fig. 3:**
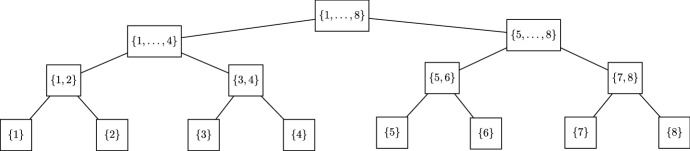
Canonical balanced dimension tree for dimension $d=8$

An example for a dimension tree with dimension $d = 8$ is shown in Fig. 3.

The rank of a representation in the hierarchical Tucker format depends on the bisection, i.e., on the dimension tree. For each node *t* there is a rank component $r_t$ corresponding to the matrix rank of the matricization $\mathsf {B}^{(t)}$. Thus, the representation rank in the hierarchical Tucker format is a tuple depending on the tree $\mathcal {T}$ and will be denoted by $\mathbf {r} := (r_t)_{t \in \mathcal {T}}$. Again, the minimal representation rank is called the *hierarchical Tucker rank*.

Given a dimension tree $\mathcal {T}$, truncation can be performed in a quasi-optimal way, cf. (Grasedyck 2010). There it is shown that the error in the $\ell _2$-norm caused by truncation of a tensor $\mathsf {B}\in \mathbb {R}^{\mathcal {I}}$ to rank $\mathbf {r}= \left( r_{t}\right) _{t \in \mathcal {T}}$ is bounded by

12 $$\begin{aligned} \Vert \mathsf {B}- {\text {trunc}}(\mathsf {B}) \Vert ^2 \le \sum \limits _{t \in \mathcal {T}} \sum \limits _{m > r_{t}} \sigma _{t,m}^2 \le ~ C d ~\Vert \mathsf {B}- \mathsf {B}^{{\text {best}}} \Vert ^2, \end{aligned}$$

where $\sigma _{t, m}$ is the *m*-th singular value of the matricization $\mathsf {B}^{\left( t\right) }$, $\mathsf {B}^{{\text {best}}}$ is a best rank-$\mathbf {r}$ approximation, and $C < 2$ is a small constant. This error bound allows for truncation with guaranteed accuracy. Thus, truncating after arithmetic operations allows us to perform iterative methods in an efficient way and preserves their convergence (Hackbusch et al. 2008).

Inspired by (Hackbusch 2012, Chapter 13), Table 1 lists some of these operations, as well as their respective cost.

**Table 1:** Operations and their cost for a *d*-dimensional tensor with representation rank bounded by *r* and mode sizes bounded by *n* in the hierarchical Tucker format

Operation	Cost	Reference
Storage	$\mathcal {O}\left( dr^3 + d n r \right)$	(Grasedyck 2010, Lemma 3.7)
Addition	$\mathcal {O}\left( d n r^2 + d r^4 \right)$	(Hackbusch 2012, 13.1.4)
Evaluation	$\mathcal {O}\left( dr^3 \right)$	(Hackbusch 2012, 13.2.3)
Inner product	$\mathcal {O}\left( dnr^2 + dr^4 \right)$	(Hackbusch 2012, Lemma 13.7)
Apply an operator	$\mathcal {O}\left( d n^2 r \right)$	(Hackbusch 2012, 13.9.1)
Truncation	$\mathcal {O}\left( dn r^2 + d r^4 \right)$	(Hackbusch 2012, (11.46*c*))

In the hierarchical Tucker format, the storage complexity grows only linearly in the dimension *d*. This allows for efficient storage and overcomes the state-space explosion provided we have low ranks. In our case the dimension of the distribution tensors is equal to the number *d* of automata, and the mode sizes are the numbers of states $n_{i}$ for each automaton $\mathcal {A}_i$.

It is possible to convert a CP representation of rank *r* easily to a hierarchical Tucker one, where all rank components are bounded by *r* independent of the dimension tree, cf. (Hackbusch 2012). In general, however, the rank in the hierarchical Tucker format depends on the selected bisection, i.e., on the dimension tree. Thus, the question arises how to choose the dimension tree in order to obtain a low rank. For further explanation on the choice of tree we refer the reader to more advanced papers (Grasedyck and Hackbusch 2011; Ballani and Grasedyck 2014). We will discuss this issue for the marginal distribution $\mathsf {p}$ in Sects. 4.2 and 4.3 using some numerical experiments.

### Related work

For large Markov chains, the data-sparse structure of the operator has already been exploited for fast matrix-vector applications (Buchholz and Dayar 2007). However, this allowed only the operator, but not the distributions, to be stored in a data-sparse form. The distributions were stored entry by entry and thus suffer from the state-space explosion. In Schill et al. (2019) a strategy based on a splitting of the operator ${\mathsf {Id}}- \mathsf {Q}= \mathbf {D} + \mathbf {L}$ in a diagonal matrix $\mathbf {D}$ and a strictly lower triangular matrix $\mathbf {L}$ was presented. Using $\mathbf {L}^{d} = 0$ and the Neumann series, the marginal distribution is given by

13 $$\begin{aligned} \mathsf {p}= \sum \limits _{k=0}^{d-1} \left( - \mathbf {D}^{-1} \cdot \mathbf {L}\right) ^k \mathbf {D}^{-1} \cdot \mathsf {p}(0). \end{aligned}$$

Note that this equation requires inverting the diagonal operator $\mathbf {D}$ or solving the corresponding linear system. In contrast to the matrix case, inverting a diagonal operator in low-rank tensor formats is not straightforward, and it is unclear whether the solution itself has a low-rank structure. Solving each linear system involving $\mathbf {D}$ would result in solving $(d+1)$ systems with a comparable complexity as the original one. Thus, the strategy in (13) cannot easily be transferred to low-rank tensors to overcome the state-space explosion.

Alternatively, Gotovos et al. (2021) recently proposed a strategy for learning the parameters of a Mutual Hazard Network without computing the marginal distribution for all states. Given a data set of observations, optimizing the log-likelihood requires the marginal probability of each state observed, which is represented as sum of probabilities for all possible transition sequences leading to this state. For an observation with *m* mutations present there are *m*! possible sequences. In order to deal with the sheer number of sequences, a stochastic approximation based on a Metropolis-Hastings algorithm is used. Instead of restricting the state space, here we want to exploit the given low-rank tensor structures of the model, cf. Sect. 2.5, to overcome the state-space explosion.

We briefly review existing strategies to compute distributions using low-rank tensor formats. The CP format has been used extensively to approximate, in particular, stationary distributions, e.g., in Kulkarni (2011), and to derive conditions for their existence, e.g., in Fourneau (2008). In Benson et al. (2017) stationary distributions for random walks are computed via an eigenvalue problem using the CP format. The tensor-train format was successfully used for the computation of, e.g., transient distributions (Johnson et al. 2010), mean times to failure (Robol and Masetti 2019), and stationary distributions (Bolten et al. 2018; Kressner and Macedo 2014). There mainly optimization-based approaches were presented. In Buchholz et al. (2016) the hierarchical Tucker format was applied to reduce the storage cost for distributions and the computational cost for performing basic operations. Continuing in Buchholz et al. (2017) adaptive truncation strategies for the computation of stationary distributions using iterative methods were presented. In Kressner and Macedo (2014) a power iteration based on a formulation of the stationary solution by an eigenvalue problem was derived, where after each application of the operator in the power iteration the current approximation was rescaled to ensure that it sums up to one.

However, the time-marginal distribution we are interested in has not yet been studied in the context of low-rank tensors. For this reason, we present such a method in the following section.

### Low-rank method

We now make use of low-rank tensor formats to approximate the marginal distribution $\mathsf {p}$ as the solution of (10). Since the exact solution $\mathsf {p}$ is a probability distribution, its entries sum up to one, i.e., it fulfills

14 $$\begin{aligned} \langle {\mathbb {1}}, \mathsf {p}\rangle = 1, \end{aligned}$$

where ${\mathbb {1}}\in \mathbb {R}^{S}$ is the tensor of all ones. To allow for a probabilistic interpretation of an approximation of $\mathsf {p}$, we need to treat (14) as an additional constraint. To this end, we now present an iterative method based on the Neumann series (Dubois et al. 1979) and the uniformization method (Grassmann 1977). The following lemma holds true for any discrete-state continuous-time Markov chain.

#### Lemma 2

Let $\mathsf {Q}\in \mathbb {R}^{S\times S}$ be any infinitesimal generator over any discrete state space $S$ and $\mathsf {p}(0)\in \mathbb {R}^{S}$ any initial distribution. For $\gamma \ge \max _{x \in S} \vert \mathsf {Q}_{x,x} \vert > 0$ the marginal distribution can be represented as a Neumann series,

15 $$\begin{aligned} \mathsf {p}= \frac{1}{1 + \gamma } ~ \sum \limits _{m = 0}^{\infty } ~ \left( \frac{\gamma }{1 + \gamma } \mathsf {P}_{\gamma }\right) ^m \mathsf {p}(0)\quad \text {with} \quad \mathsf {P}_{\gamma }:= {\mathsf {Id}}+ \frac{1}{\gamma } \mathsf {Q}, \end{aligned}$$

and the series converges.

#### Proof

Due to Lemma 1 the marginal distribution can be represented as

$$\begin{aligned} \mathsf {p}= \left( {\mathsf {Id}}- \mathsf {Q}\right) ^{-1} \mathsf {p}(0)= \frac{1}{1 + \gamma } \left( {\mathsf {Id}}- \left( \frac{\gamma }{1 + \gamma } {\mathsf {Id}}+ \frac{1}{1 + \gamma } \mathsf {Q}\right) \right) ^{-1} \mathsf {p}(0). \end{aligned}$$

In order to use the Neumann series for inverting ${\mathsf {Id}}- \left( \frac{\gamma }{1 + \gamma } {\mathsf {Id}}+ \frac{1}{1 + \gamma } \mathsf {Q}\right) =: {\mathsf {Id}}- \mathbf {G}_\gamma$, it is sufficient to show that the spectral radius $\rho \left( \mathbf {G}_\gamma \right) < 1$. Applying the Gershgorin circle theorem (Gershgorin 1931) leads to

$$\begin{aligned} \sigma \left( \mathbf {G}_\gamma \right) \subseteq \bigcup \limits _{x \in S} \underbrace{\Big \{ z \in \mathbb {C}~\Big \vert ~ \Big \vert z - \frac{\gamma - \vert \mathsf {Q}_{x,x}\vert }{1 + \gamma } \Big \vert \le \frac{\vert \mathsf {Q}_{x,x} \vert }{1 + \gamma } \Big \} }_{=: Z_{x, \gamma } }. \end{aligned}$$

Thus, the spectral radius is bounded by

$$\begin{aligned} \rho \left( \mathbf {G}_\gamma \right) = \max \limits _{z \in \sigma \left( \mathbf {G}_\gamma \right) } \vert z \vert \le \max \limits _{x \in S} \left( \max \limits _{z \in Z{x, \gamma }} \vert z \vert \right) . \end{aligned}$$

For $x \in S$ and $z \in Z_{x, \gamma }$ we obtain using $\gamma \ge \vert \mathsf {Q}_{x,x}\vert$:

$$\begin{aligned} \Big \vert \vert z \vert - \underbrace{\frac{\gamma - \vert \mathsf {Q}_{x,x}\vert }{1 + \gamma } }_{ \ge 0} \Big \vert {\mathop {\le }\limits ^{\triangledown }} \Big \vert z - \frac{\gamma - \vert \mathsf {Q}_{x,x}\vert }{1 + \gamma } \Big \vert \le \frac{\vert \mathsf {Q}_{x,x} \vert }{1 + \gamma } \quad \Rightarrow \quad \vert z \vert \le \frac{\gamma }{1 + \gamma } < 1. \end{aligned}$$

Since the spectral radius is smaller than 1, the Neumann series converges with

$$\begin{aligned} \mathsf {p}= \frac{1}{1 + \gamma } \sum \limits _{m = 0}^\infty \mathbf {G}_\gamma ^m \mathsf {p}(0)= \frac{1}{1 + \gamma } ~ \sum \limits _{m = 0}^{\infty } ~ \left( \frac{\gamma }{1 + \gamma } \mathsf {P}_{\gamma }\right) ^m \mathsf {p}(0). \end{aligned}$$

$$\square $$

Alternatively we can derive (15) using the uniformization method. Here, the idea is to describe a continuous-time Markov chain by a discrete-time Markov chain with a time increment that is exponentially distributed. With this interpretation, we write the time-dependent probability distribution in (1) as

$$\begin{aligned} \mathsf {p}(t) = \sum \limits _{m = 0}^{\infty } \frac{\left( \gamma t\right) ^m}{m!} \exp (-\gamma t) \left( \mathsf {P}_{\gamma }\right) ^m \mathsf {p}(0)\end{aligned}$$

for a time $t \ge 0$. Note that $\mathsf {P}_{\gamma }$ is the transition probability matrix of a discrete-time Markov chain, since $\gamma$ is a bound on the diagonal entries of $\mathsf {Q}$. Marginalization of time similar to (2) and substitution leads to (15):

$$\begin{aligned} \mathsf {p}&= \int \limits _{0}^{\infty } \exp (-t) ~ \mathsf {p}(t) ~\mathrm {d}t ~=~ \sum \limits _{m = 0}^{\infty } ~ \frac{\gamma ^m}{m!} ~\int \limits _{0}^{\infty } t^m ~ \exp (- (\gamma + 1 ) t) ~\mathrm {d}t ~ \left( \mathsf {P}_{\gamma }\right) ^m \mathsf {p}(0)\\&= \sum \limits _{m = 0}^{\infty } ~ \frac{\gamma ^m ~ \varGamma (m + 1 )}{ m! \left( 1 + \gamma \right) ^{m+1}} ~ \left( \mathsf {P}_{\gamma }\right) ^m \mathsf {p}(0)~=~ \frac{1}{1 + \gamma } ~ \sum \limits _{m = 0}^{\infty } ~ \left( \frac{\gamma }{1 + \gamma } ~\mathsf {P}_{\gamma }\right) ^m \mathsf {p}(0), \end{aligned}$$

where $\varGamma$ denotes the gamma function.

We now discuss approximation strategies for (15). A natural approximation would be

16 $$\begin{aligned} \tilde{\mathsf {p}}^{(k)} := \frac{1}{1 + \gamma } ~ \sum \limits _{m = 0}^{k} ~ \left( \frac{\gamma }{1 + \gamma } ~\mathsf {P}_{\gamma }\right) ^m \mathsf {p}(0)\end{aligned}$$

for $k \in \mathbb {N}$ which is an approximation from below (entry by entry), i.e., $\tilde{\mathsf {p}}^{(k)} _x \le \mathsf {p}_x$ for all $x \in S$ and all $k \in \mathbb {N}$. Based on the properties of the Neumann series this sequence converges linearly to $\mathsf {p}$, but $\tilde{\mathsf {p}}^{(k)}$ satisfies the normalization condition (14) only approximately for $k \rightarrow \infty$. As $\mathsf {P}_{\gamma }$ is a transition probability matrix, its application to a probability distribution leads to a probability distribution again satisfying (14), and thus

17 $$\begin{aligned} \langle {\mathbb {1}}, \sum \limits _{m = 0}^{k} ~ \left( \frac{\gamma }{1 + \gamma } ~ \mathsf {P}_{\gamma }\right) ^m \mathsf {p}(0)\rangle = \sum \limits _{m = 0}^{k} ~ \left( \frac{\gamma }{1 + \gamma }\right) ^m = \frac{\left( 1 + \gamma \right) ^{k + 1} - \gamma ^{k+1}}{\left( 1 + \gamma \right) ^k} \end{aligned}$$

for all $k \in \mathbb {N}$. By scaling each element of the sequence, we obtain

18 $$\begin{aligned} \mathsf {p}^{(k)}:= \frac{\left( 1 + \gamma \right) ^k}{\left( 1 + \gamma \right) ^{k + 1} - \gamma ^{k+1}} ~ \sum \limits _{m = 0}^{k} ~ \left( \frac{\gamma }{1 + \gamma }~ \mathsf {P}_{\gamma }\right) ^m \mathsf {p}(0)\end{aligned}$$

for $k \in \mathbb {N}$, which is now an approximation to $\mathsf {p}$ that satisfies (14). We prove its linear convergence to $\mathsf {p}$ in the following theorem for any discrete-state continuous-time Markov chain.

#### Theorem 1

Let $\mathsf {Q}\in \mathbb {R}^{S\times S}$ be any infinitesimal generator over any discrete state space $S$ and $\mathsf {p}(0)\in \mathbb {R}^{S}$ any initial distribution. Let further $\mathsf {p}$ be the solution of the corresponding linear system (10), and $\mathsf {p}^{(k)}$ be defined by (18) for all $k \in \mathbb {N}$. Then $\mathsf {p}^{(k)}$ converges linearly to $\mathsf {p}$ as *k* approaches infinity, i.e.,

$$\begin{aligned} \lim \limits _{k \rightarrow \infty } \mathsf {p}^{(k)}= \mathsf {p}\qquad \text {with} \qquad \big \Vert \mathsf {p}^{(k)}- \mathsf {p}\big \Vert \le c \cdot \biggl (\frac{\gamma }{1 + \gamma }\biggr )^k \quad \text {for all } k \in \mathbb {N}, \end{aligned}$$

where $$c =\big \Vert \frac{1}{1 + \gamma } \mathsf {p}(0)- \mathsf {p}\big \Vert + \gamma \Vert \mathsf {p}\Vert $$.

#### Proof

For $\alpha _k := \frac{(1 + \gamma )^k}{(1 + \gamma )^{k + 1} - \gamma ^{k+1}}$, $\alpha := \frac{1}{1+\gamma }$ and any $k \in \mathbb {N}$ we have

$$\begin{aligned} \vert \alpha _k - \alpha \vert&= \bigg \vert \frac{ \gamma ^{k+1}}{(1+\gamma ) ((1+\gamma )^{k+1} - \gamma ^{k+1})} \bigg \vert< \frac{\gamma }{1+\gamma } ~ \vert \alpha _{k-1} - \alpha \vert \\&< \biggl (\frac{\gamma }{1 + \gamma }\biggr )^k ~ \vert \alpha _0 - \alpha \vert = \biggl (\frac{\gamma }{1 + \gamma }\biggr )^{k+1}. \end{aligned}$$

Furthermore we obtain

$$\begin{aligned} \big \Vert \mathsf {p}^{(k)}- \mathsf {p}\big \Vert&\le \big \Vert \mathsf {p}^{(k)}- \tilde{\mathsf {p}}^{(k)} \big \Vert + \big \Vert \tilde{\mathsf {p}}^{(k)} - \mathsf {p}\big \Vert \\&\le \vert \alpha _k - \alpha \vert \bigg \Vert \sum \limits _{m = 0}^{k} ~ \biggl (\frac{\gamma }{1 + \gamma }~\mathsf {P}_{\gamma }\biggr )^m \mathsf {p}(0)\bigg \Vert + \biggl ( \frac{\gamma }{1+\gamma }\biggl )^k \Vert \alpha \mathsf {p}(0)- \mathsf {p}\Vert \\&\le \bigl ( \Vert \alpha \mathsf {p}(0)- \mathsf {p}\Vert + \gamma \Vert \mathsf {p}\Vert \bigr ) \cdot \biggl (\frac{\gamma }{1 + \gamma }\biggr )^k. \end{aligned}$$

Since $\frac{\gamma }{1 + \gamma } < 1$, the linear convergence follows.

So far, we have not needed any additional assumptions on the Markov chain. However, we are particularly interested in high-dimensional problems and want to overcome the state-space explosion using low-rank tensor methods. Therefore, we now assume that both, the generator $\mathsf {Q}$ and the initial distribution $\mathsf {p}(0)$, have a low-rank tensor representation, as derived in Sect. 2. In order to employ low-rank tensor formats, we supplement the iterative method corresponding to (18) by truncation. As shown in Hackbusch et al. (2008), a convergent iterative method combined with truncation still converges if the truncation error is sufficiently small. However, the truncation could change the normalization of the iteration. Therefore, we replace the scaling with $\alpha _k$ by dividing by $\langle {\mathbb {1}}, \mathsf {p}^{(k)} \rangle$ as shown in Algorithm 1. The procedure should be continued until the norm of the relative residual is smaller than a given tolerance $\text {tol}>0$.
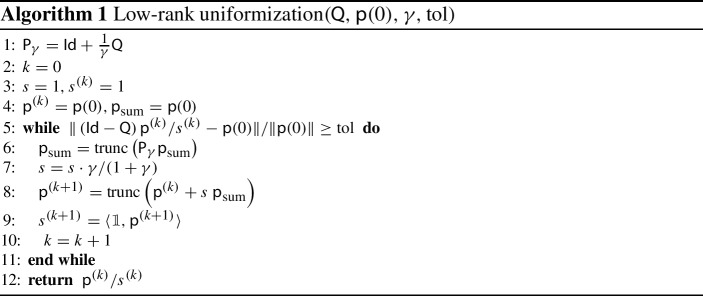


Note that when using the unnormalized approximation $\tilde{\mathsf {p}}^{(k)}$ by replacing the normalization by $s^{(k)}$ with $\left( 1 + \gamma \right)$, the norm of the relative residual turns out not to be a useful measure and one must also consider the distance of $s^{(k)}$ and $\left( 1 + \gamma \right)$. By taking the relative residual of the normalized version, this is done implicitly.

Our sequence $\left( \mathsf {p}^{(k)}\right) _k$ is defined as a normalized version of $\left( \tilde{\mathsf {p}}^{(k)} \right) _k$, which is an approximation to $\mathsf {p}$ from below (entry-by-entry). By estimating the Poisson distributed error of $\tilde{\mathsf {p}}^{(k)}$, one can obtain

$$\begin{aligned} {\hat{\mathsf {p}}}^{(k)} := \tilde{\mathsf {p}}^{(k)} + \left( 1 - \sum \limits _{m=0}^k \frac{\gamma ^m}{(1 + \gamma )^{m+1}}\right) {\mathbb {1}}\end{aligned}$$

for $k \in \mathbb {N}$ as unnormalized approximation to $\mathsf {p}$ from above (entry-by-entry) which also converges linearly to $\mathsf {p}$. In numerical experiments (not further discussed here), ${\hat{\mathsf {p}}}^{(k)}$ and also its normalized version turned out to be unpromising, since significantly more iteration steps were required to achieve the same approximation quality measured by the relative residual.

In general, Algorithm (I) does not require assumptions  or (II) but $\mathsf {Q}$ and $\mathsf {p}(0)$ to have appropriate low-rank tensor representations and an upper bound $\gamma \ge \max _x \vert \mathsf {Q}_{x,x}\vert$. In general, computing all diagonal entries of $\mathsf {Q}$ is in $\mathcal {O}(n^d)$. Nevertheless, here we focus on Markov chains satisfying (I) and (II) with an infinitesimal generator $\mathsf {Q}$ in (9) depending on parameters $\mathsf {\Theta }$. This allows for the following inexpensive upper bound:

19 $$\begin{aligned} \gamma = \sum \limits _{i = 1}^d ~ \max \limits _{x_{i} \in S_{i}} \left( \sum \limits _{\begin{array}{c} t_i \in T_{i}\\ t_i = (x_i \rightarrow y_i) \end{array}} ~ \prod \limits _{j = 1}^d ~ \max \limits _{x_{j} \in S_j} ~ \mathsf {\Theta }(t_i, x_j) \right) , \end{aligned}$$

which can be computed in $\mathcal {O}\left( d^2 n^2 T\right)$,^3^ where $T = \max _{i} \vert T_{i} \vert$ is the maximum number of possible transitions in an automaton. In the case of strongly varying parameters $\mathsf {\Theta }$, (19) may be a significant overestimation for the diagonal entries of $\mathsf {Q}$. The question of how to determine a tighter bound with polynomial effort using low-rank tensor formats will be dealt with in future work.

## Numerical experiments

We illustrate our method for the computation of time-marginal distributions in numerical experiments based on the model of Mutual Hazard Networks with *d* automata (genomic events). In this case, the parameters can be summarized in a matrix $\mathbf {\mathsf {\Theta }} \in \mathbb {R}^{d \times d}$.

### Setting

#### Construction of synthetic parameters

We consider *d* automata and parameters $\mathsf {\Theta }\in \mathbb {R}_{>0}^{d \times d}$ with a particular block-diagonal form. Each quadratic block of size $b \times b$ characterizes a subset of *b* automata which directly affect one another. We draw all within each block so that their logarithmic values are normally distributed with mean $\mu = 0$ and standard deviation $\sigma = 0.25$. Unless stated otherwise, all other parameters are exactly 1 (logarithmic values of 0), which corresponds to a neutral direct effect between automata of different blocks. Based on these blocks we study three types of parameters:

1. *(Strict) block structure*: There are direct effects, i.e., parameters $\mathsf {\Theta }_{i,j} \ne 1$, only between automata in the same block.
2. *Neighbor block structure*: In addition to the effects within each block in (B1), there are direct effects between randomly chosen automata in neighboring blocks. The parameters $\mathsf {\Theta }_{i,j}$ are drawn such that their logarithmic values $\log \left( \mathsf {\Theta }_{i,j}\right)$ are normal distributed with mean 0 and $\sigma = 0.125$ and the automata (*i*, *j*) are uniformly randomized in neighboring blocks. For each pair of neighboring blocks 4 random effects.
3. *Neighbor block structure with additional random effects*: Besides the effects in (B2), there are direct effects between randomly chosen automata of different blocks. Again the corresponding parameters $\mathsf {\Theta }_{i,j}$ are drawn such that their logarithmic values $\log \left( \mathsf {\Theta }_{i,j}\right)$ are normal distributed with mean 0 and $\sigma = 0.125$. The choice of (*i*, *j*) is also uniformly randomized. For each parameter matrix $\mathsf {\Theta }\in \mathbb {R}_{> 0}^{d \times d}$ we add 8 random effects.

The blocks in (B1) correspond to non-interacting subsystems of the process. In biological models, these often represent well-defined, distinct pathways of genes that regulate specific functions of the cell such as the cell cycle, apoptosis and growth (Sanchez-Vega et al. 2018). Note that even if subsystems do not interact, it is not possible to trivially express the solution as a Kronecker product of solutions per block, since correlations are induced by the observation time acting as a confounding variable, see also (Gotovos et al. 2021, Section 3.2). Finding such blocks or groupings, e.g., during parameter determination, can be important for understanding and treating tumors. However, in practice, the behavior of a biological system can rarely be separated into well-defined subsystems that do not interact, so (B1) is an oversimplification. Hence, we add interactions between different subsystems in (B2) and (B3). For parameters of type (B2) all automata or events already interact indirectly. For example, if event $\mathcal {A}_1$ favors the occurrence of event $\mathcal {A}_2$, i.e., $\mathsf {\Theta }_{2, 1} > 1$, and at the same time event $\mathcal {A}_2$ inhibits the occurrence of event $\mathcal {A}_3$, i.e., $\mathsf {\Theta }_{3,2} < 1$, then event $\mathcal {A}_1$ also indirectly inhibits event $\mathcal {A}_3$ even if the direct effect of $\mathcal {A}_1$ on $\mathcal {A}_3$ is neutral. We reduce the standard deviation when sampling the effects between different blocks to simulate that the influences between different blocks are much smaller than those within.

#### Default settings for the algorithm

For the application of Algorithm 1 we always choose the bound $\gamma$ as in (19), which for Mutual Hazard Networks can be reduced to

20 $$\begin{aligned} \gamma = \sum \limits _{i = 1}^d ~ \prod \limits _{j = 1}^d \max \{1, \mathbf {\mathsf {\Theta }}_{i, j}\}. \end{aligned}$$

**Fig. 4:**
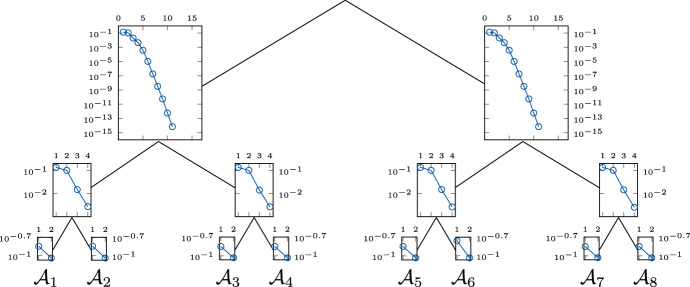
Mean of singular values of matricizations of $\mathsf {p}$ for the canonical balanced tree and 100 sample parameters of type (B1) with $d=8$ automata and 2 blocks of size $b=4$ each

Unless stated otherwise, we use a canonical balanced dimension tree for the application of the hierarchical Tucker format, i.e., the automata are assigned to the leaves following their ordering, see Fig. 4. We perform all experiments for 100 randomly generated sample parameters for each combination parameter type, number of blocks and number *d* of automata. We compute low-rank approximations of the marginal distribution $\mathsf {p}$ using Algorithm 1 with a maximum relative truncation error ${ \Vert \mathsf {B}- {\text {trunc}}(\mathsf {B}) \Vert }/{\Vert \mathsf {B}\Vert } \le \varepsilon _{\text {trunc}}= 10^{-7}$, see (12). The algorithm stops when the norm of the relative residual is smaller than a tolerance value of $\text {tol}= 10^{-4}$. The mean values we compute are arithmetic means. In the following, we call the representation rank of this approximation tensor an *approximation rank* of $\mathsf {p}$.

### Study of singular values

One fundamental assumption for solving linear systems using low-rank tensor methods is that a solution can be well approximated by a tensor of low rank. According to the error bound in (12), the truncation error is determined by the singular values of the matricization corresponding to the chosen dimension tree. A fast decay of those singular values indicates that a tensor can be approximated accurately by one of low rank.

To analyze this issue, we solve (10) using classical matrix methods of MATLAB (Natick 2019) and compute the singular values of the corresponding matricization using the htucker toolbox (Kressner and Tobler 2014). Figure 4 shows the decay of the singular values of each matricization corresponding to the canonical balanced tree for $d = 8$ automata and parameters of type (B1) with 2 blocks of size $b = 4$ each. For each node the semi-logarithmic plot displays the means of the singular values over 100 samples.

We observe that the singular values exhibit an exponential decay. The two matricizations closest to the root are transposes of each other, and therefore their singular values are identical. The smallest 5 of the 16 singular values are indistinguishable from zero and therefore cannot be displayed in the semi-logarithmic plot. For other standard deviation $\sigma$, we observed a similar drop in singular values (not shown here). The exponential decay of the singular values indicates that the marginal distribution $\mathsf {p}$ can be well approximated with low rank.

### Study of the tree structure

In general, the rank in the hierarchical Tucker format depends on the structure of the tree. In the following, we study how the ordering of the automata in the leaves of the tree affects the low-rank approximability. We preserve the balanced binary structure of the tree because this is advantageous for parallelization, cf. (Grasedyck and Löbbert 2018).

**Fig. 5:**
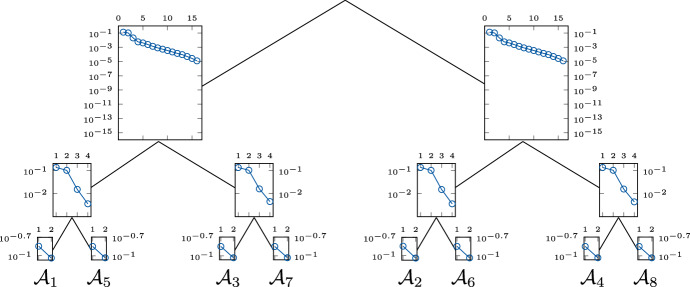
Mean of singular values of matricizations of $\mathsf {p}$ for a modified balanced tree and 100 sample parameters of type (B1) with $d=8$ automata and 2 blocks of size $b=4$ each

We already studied the singular values of the matricizations corresponding to the canonical balanced tree, see Fig. 4. We now change only the arrangement of the automata in the leaves of the tree and compute the marginal distribution $\mathsf {p}$ again. The decay of the singular values for each matricization is shown in Fig. 5, where the semi-logarithmic plot at each vertex displays the means of the singular values over 100 samples.

Comparing Figs. 4 and 5, we observe that the choice of the canonical dimension tree, i.e., the original ordering in the leaves, results in a significantly faster decay of the singular values close to the root. Figure 5 shows that the singular values closest to the root also have an exponential decrease, but at a much slower rate. Note that 2 blocks of size $b = 4$ imply that the automata $\{\mathcal {A}_1, \mathcal {A}_2, \mathcal {A}_3, \mathcal {A}_4\}$ interact directly with one another. The same holds for the automata $\{\mathcal {A}_5, \mathcal {A}_6, \mathcal {A}_7, \mathcal {A}_8\}$. There are no direct interactions between automata of different blocks. Hence, the modified balanced tree separates strongly interacting automata, which explains the slower decay of the singular values in level 1 (level 0 being the root). In contrast, the canonical balanced tree separates weakly interacting automata, leading to a faster decay of the singular values. We will make similar observations when studying the approximation rank. Based on this observation, the question arises whether and how a suitable dimension tree can be determined. Ballani and Grasedyck (2014) developed a black box method to determine a dimension tree for general low-rank problems. In our concrete model problem, however, the parameters $\mathsf {\Theta }$ already seem to give a-priori information about suitable structures. How exactly a dimension tree can be constructed based on the parameters is, however, not a trivial question and requires a more detailed investigation, which would exceed the scope of this paper. In the following, we will focus on the investigation of the presented method.

### Comparison with a matrix-based method

We check our method in Algorithm 1 against a classical method based on matrix operations presented in Schill et al. (2019), see (15). To do so, we use Mutual Hazard Networks computed there and the corresponding infinitesimal generators $\mathsf {Q}$. The parameters are optimized based on data for breast cancer, colorectal cancer, renal cell carcinoma and glioblastoma which represent mutual effects between $d=10, 11, 12$ and 20 genomic events each. The specific parameters and their interpretation can be found in Schill et al. (2019) and its supplementary material. The network for breast cancer is also shown in Fig. 1. We use the settings in Sect. 4.1.2 for Algorithm 1.

We compare the result $\mathsf {p}_{\text {tensor}}$ of Algorithm 1 using tensor methods and $\mathsf {p}_{\text {vector}}$ of (13) based on matrix operations with respect to the relative residual ${\Vert ({\mathsf {Id}}- \mathsf {Q}) \mathsf {p}- \mathsf {p}(0)\Vert }/{\Vert \mathsf {p}(0)\Vert }$ and the distance of both results. We measure the distance using the relative Euclidean distance ${ \Vert \mathsf {p}_{\text {vector}} - \mathsf {p}_{\text {tensor}} \Vert }/{\Vert \mathsf {p}_{\text {vector}} \Vert }$ and the *Kullback Leibler (KL) divergence* (Kullback 1997) from $\mathsf {p}_{\text {tensor}}$ to $\mathsf {p}_{\text {vector}}$. The KL divergence from $\mathsf {p}_{\text {tensor}}$ to $\mathsf {p}_{\text {vector}}$ is defined as

21 $$\begin{aligned} {\text {KL}}\left( {\mathsf {p}_{\text {vector}}, \mathsf {p}_{\text {tensor}}}\right) = \sum _x (\mathsf {p}_{\text {vector}})_x \cdot \log \frac{(\mathsf {p}_{\text {vector}})_x}{(\mathsf {p}_{\text {tensor}})_x} \end{aligned}$$

and measures how $\mathsf {p}_{\text {tensor}}$ differs from $\mathsf {p}_{\text {vector}}$ with respect to $\mathsf {p}_{\text {vector}}$. Since $\mathsf {p}_{\text {tensor}}$ is only an approximation of the marginal distribution $\mathsf {p}$, there might be some negative values which are absolutely small. To deal with this problem, we use the convention $0 \cdot \log (0) = 0$ and cut off $(\mathsf {p}_{\text {vector}})_x \le \varepsilon _{\text {cut-off}} = 10^{-8}$ to zero. Besides, we list the costs for storing $\mathsf {p}_{\text {vector}}$ and $\mathsf {p}_{\text {tensor}}$, respectively, in values which have to be stored for each solution. Table 2 summarizes the values for four different Mutual Hazard Networks corresponding to different types of tumors.

**Table 2:** Comparison of the results $\mathsf {p}_{\text {tensor}}$ of Algorithm 1 based on low-rank tensors and $\mathsf {p}_{\text {vector}}$ of (13) based on matrix operations for different Mutual Hazard Networks from Schill et al. (2019)

Disease	*d*	Relative residual $\mathsf {p}_{\text {vector}}$	Relative residual $\mathsf {p}_{\text {tensor}}$	Relative Euclidean distance	Absolute KL divergence	Storage cost $\mathsf {p}_{\text {vector}}$	Storage cost $\mathsf {p}_{\text {tensor}}$
Breast cancer	10	1.7e$-$17	8.8e$-$05	3.3e$-$04	3.3e$-$07	1024	3052
Colorectal cancer	11	2.9e$-$17	9.8e$-$05	3.5e$-$04	3.1e$-$07	2048	4396
Renal cell carcinoma	12	1.1e$-$17	9.8e$-$05	1.2e$-$04	1.4e$-$06	4096	4553
Glioblastoma	20	1.6e$-$17	9.7e$-$05	9.7e$-$05	4.4e$-$06	1,048,576	44,065

By construction, the residuals for $\mathsf {p}_{\text {tensor}}$ of Algorithm 1 are slightly below the requested tolerance $\text {tol}= 10^{-4}$. For smaller values of $\text {tol}$ (not shown in Table 2) smaller relative residuals for $\mathsf {p}_{\text {tensor}}$ can be reached by increasing the number of iteration steps. The results $\mathsf {p}_{\text {vector}}$ of (13) solve the equation nearly exactly. The relative Euclidean distances as well as the KL divergences from $\mathsf {p}_{\text {tensor}}$ to $\mathsf {p}_{\text {vector}}$ have very small absolute values. This implies that both results describe nearly the same distribution. Calculating the KL divergences for smaller cut-off parameters $\varepsilon _{\text {cut-off}} \in \{10^{-9}, \dots , 10^{-16}\}$, we obtain the same values for the first three networks as with $\varepsilon _{\text {cut-off}} = 10^{-8}$. For the last (and largest) network, if $\varepsilon _{\text {cut-off}} \le 10^{-10}$, we observe some negative entries with very small absolute values, i.e., $\vert (\mathsf {p}_{\text {tensor}})_x \vert \le 10^{-9}$. This can be explained by the truncation used in Algorithm 1 to reduce the storage and computational complexity. Hence, we need $\varepsilon _{\text {cut-off}} > 10^{-10}$ for this network. Calculating KL divergences requires the explicit evaluation of all entries of the tensors $\mathsf {p}_{\text {tensor}}$, since an efficient method for the entry-wise application of the logarithm within low-rank tensor formats is unknown. This limits the computation of KL divergences to tensors of small dimension *d*. Comparing the storage costs for $\mathsf {p}_{\text {vector}}$ and $\mathsf {p}_{\text {tensor}}$, we observe an overhead of storing the tensor solution compared to the vector one for diseases with $d \le 12$. However, already for $d=20$ the storage cost for the vector solution explodes, whereas the cost for the tensor solution remains moderate with about $4\%$ of the cost for $\mathsf {p}_{\text {vector}}$.

**Fig. 6:**
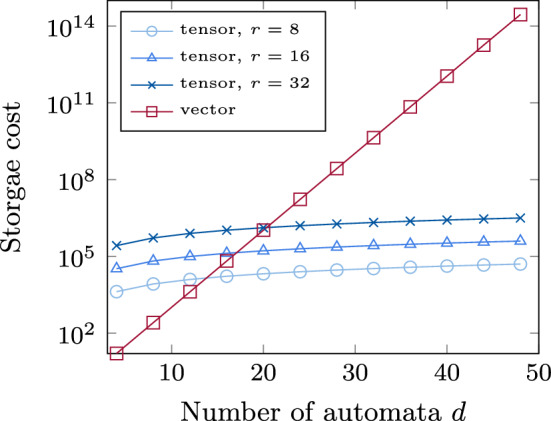
Cost for storing the marginal distribution of a Mutual Hazard Network with *d* automata and $n=2$ as a vector compared to a hierarchical Tucker (HT) tensor with constant rank $r = 8, 16, 32$

In Fig. 6, we further modeled the storage requirements for potential vector and tensor solutions as a function of *d* up to 50 in a semi-logarithmic plot. For this purpose, we made the simplified assumption of constant ranks $\mathbf {r}= (r, \dots , r)$ for the hierarchical Tucker representation of $\mathsf {p}_{\text {tensor}}$ with $r \in \{8, 16, 32\}$.

Again, we note an overhead of storing the tensor compared to the vector representation for $d \le 20$, but for $d > 20$ the tensor representation is preferable. Figure 6 shows the exponential increase of storage cost for the vector representation in the number of automata, i.e., the state-space explosion. In contrast the cost for the hierarchical Tucker representation grows only linearly in *d* as expected from Table 1. A low representation rank is essential in order to overcome the state space explosion, as it enters cubically into the storage cost. We will now investigate this in more detail.

### Study of the approximation rank

The rank $\mathbf {r}= \left( r_t\right) _{t \in \mathcal {T}}$ in a hierarchical Tucker format is a tuple depending on the underlying tree $\mathcal {T}$. For a simple comparison of tensor representations, we consider the *effective rank* $r_{{\text {eff}} }$, which we define such that the storage cost for the representation equals the cost to store one with rank $\mathbf {r}= \left( r_{{\text {eff}} }\right) _{t \in \mathcal {T}}$. In doing so, we round $r_{{\text {eff}} }$ up to the nearest integer.

**Fig. 7:**
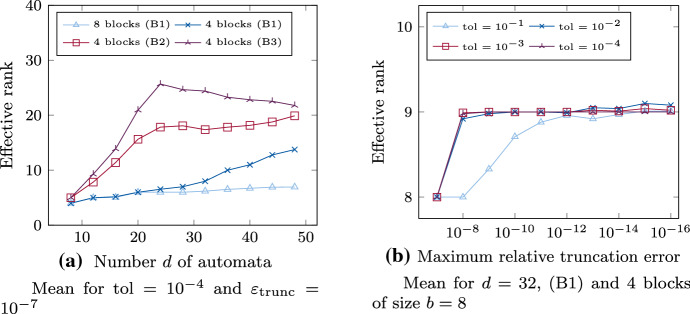
Mean of effective approximation rank of $\mathsf {p}$ as a function of the number *d* of automata in Fig. 7a and as a function of the maximum relative truncation error $\varepsilon _{\text {trunc}}$ in Fig. 7b using Algorithm 1 with tolerance $\text {tol}$ for 100 sample parameters

We compute low-rank tensor approximations of the marginal distribution $\mathsf {p}$ using Algorithm 1 as described in Sect. 4.1. We plot the effective ranks as a function of the number *d* of automata for fixed maximum relative truncation error $\varepsilon _{\text {trunc}}= 10^{-7}$ and fixed tolerance $\text {tol}= 10^{-4}$ in Fig. 7a, and as a function of $\varepsilon _{\text {trunc}}$ for fixed *d* in Fig. 7b. Both plots show mean values of effective ranks for 100 samples of parameters $\mathsf {\Theta }$ each. (In both plots the statistical errors, i.e., the variations between samples, are very small and therefore not shown.)

Figure 7a displays effective ranks for parameters $\mathsf {\Theta }$ of the three different types. The parameters of type (B1) have 8 and 4 blocks, respectively, and those types (B2) and (B3) have 4 blocks. The size of each block increases in the number *d* of automata, i.e., for 8 blocks the size of each block is $b \in \{1, \dots , 6\}$ and for 4 blocks it is $b \in \{2, \dots , 12\}$. The automata belonging to a block are each grouped by the tree structure as in Fig. 4.

We observe that $\mathsf {p}$ is approximated to a tolerance of $\text {tol}= 10^{-4}$ with low rank in all cases. For separated blocks of type (B1), drawn in blue, the effective rank increases slightly in the number *d* of automata. Since the number of blocks is kept constant, also the size of each block increases in *d*. For this reason, especially for 4 blocks, there is a superposition of the effects of increasing d and increasing block size on the effective rank. This becomes particularly clear in the comparison between 8 and 4 blocks per parameter. In case of 8 blocks, the effective rank increases much more flatly and seems to be almost independent of the number *d* of automata constrained by $r_{{\text {eff}} }\approx 8$. The larger 4 blocks always combine the automata of two smaller blocks, i.e., they are twice as large. We thus hold that although the approximation ranks increase slightly with the number *d* of automata as well as the number *b* of directly interacting automata, they remain comparatively small, provided that the partitioning in the dimensional tree is preserved. As in Fig. 5, separating the automata of a block in the dimension tree can lead to an increase in the approximation rank (not shown here). A similar increase can be observed when further effects are added between neighboring blocks, i.e., (B2), or between randomly chosen blocks, i.e., (B3). In Fig. 7a the effective ranks for parameters with additional random effects, drawn in red, both increase in *d* until 24 and then remain almost constant at around $r_{{\text {eff}} }\approx 20$ for (B2) or slightly decreases respectively for (B3). This suggests that effects that do not fit the structure of the dimension tree can lead to an increase in the approximation rank. In addition, direct effects between neighboring blocks appear to have less impact on the approximation rank than those between more distant ones. For parameters of type (B3), we observe more significant differences between effective ranks for different samples. For some, the effective ranks are significantly above the mean, for some below it. This supports the conjecture that the location (*i*, *j*) of the direct effects $\mathsf {\Theta }_{i,j} \ne 1$ affects the approximation rank. However, if only a few of these effects are present, the marginal distribution can still be approximated with comparatively low approximation rank. These results suggest that neither the number *d* of automata nor the size of the blocks alone are responsible for an increase in rank, but that especially the distribution of the automata in the dimension tree has a large effect. How to construct an appropriate tree in order to keep ranks low using a-priori information on the parameters is a topic of ongoing research.

The semi-logarithmic plot in Fig. 7b displays effective ranks for parameters $\mathsf {\Theta }$ of type (B1) for $d=32$ automata and 4 blocks of size $b= 8$ as a function of the maximum relative truncation error $\varepsilon _{\text {trunc}}$ for different tolerances $\text {tol}= 10^{-1},\dots , 10^{-4}$. As the tolerance is lowered, the approximation of $\mathsf {p}$ corresponds to a particular stage during the iteration. We observe that all effective ranks are nearly constant in the maximum relative truncation error and the tolerance for $\text {tol}$. For $\text {tol}=10^{-1}$, the effective rank is slightly lower and for all other tolerance values it is about $r_{{\text {eff}} }\approx 9$. This observation suggests, on the one hand, that $\mathsf {p}$ can be accurately approximated with a tensor of effective rank $r_{{\text {eff}} }\approx 9$, since a more accurate truncation, i.e., smaller $\varepsilon _{\text {trunc}}$, has only very small impact on the approximation ranks. On the other hand, the fact that the ranks are low and nearly independent of the tolerance value indicates that the ranks during the iteration are also low. This allows not only for efficient storage of the resulting approximation but also for efficient computation using Algorithm 1. For parameters of types (B2) and (B3), we observed that the increase in the approximation rank is further amplified for smaller maximum relative truncation errors $\varepsilon _{\text {trunc}}$. Therefore, a suitable truncation accuracy seems to become more important for parameters with more direct effects between blocks. For Fig. 7a, the maximum relative truncation error $\varepsilon _{\text {trunc}}= 10^{-7}$ is chosen to be comparatively small, but the approximation ranks remain relatively small for all numbers *d* of automata and types of parameters. During the iteration itself, the effective ranks remained almost constant (after a start phase) similar as shown in Fig. 7b.

### Study of the speed of convergence

Having studied the low-rank structure of the distribution numerically, we now consider the convergence speed of our method. In Theorem 1 we proved that the iteration sequence converges linearly to the marginal distribution. If the truncation error is small enough, then the convergence of the method combined with truncation is preserved, cf. (Hackbusch et al. 2008). We will now see that this theoretical result also holds in practice.

**Fig. 8:**
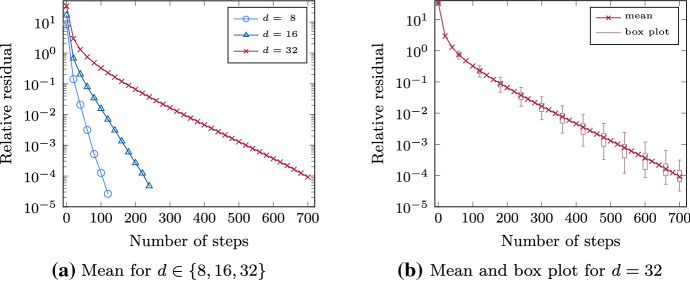
Norm of the relative residual as a function of the number of iteration steps using Algorithm 1 for 100 sample parameters of type (B1) with *d* automata and 4 blocks

To do this, we look at the decay of the relative residual as a function of the iteration steps. Again we use 100 parameter samples of type (B1) with 4 blocks for $d = 8, 16$ and 32 automata each. Figure 8a and b show semi-logarithmic plots of the norm of the relative residual as a function of the iteration step. Figure 8a displays the mean value of the relative residual and Fig. 8b additionally the corresponding box plot illustrating the variances for $d = 32$ automata.

We observe a linear convergence of the method for all values of *d*. For larger number *d* of automata the convergence slows down. A similar behavior can be observed for larger standard deviations $\sigma$ of the logarithmic parameters $\log \left( \mathsf {\Theta }_{i,j}\right)$, where a larger $\sigma$ means that also the parameters $\mathsf {\Theta }_{i,j}$ are more widely dispersed around 1. Both observations can be explained by the fact that $\gamma$ (and our upper bound in (20)) increases in *d* and $\mathsf {\Theta }_{i,j} > 1$, and thus the bound $\frac{\gamma }{1 + \gamma }$ on the convergence rate is closer to one, cf. Theorem 1. In Fig. 8b the ranges for $d=32$ given by the boxes are small, which indicates that there are only a few outliers given by the whiskers. We have confirmed our results using different numbers *d* of automata and blocks (not shown). In our tests we observe that the number of iteration steps needed to achieve a certain tolerance grows linearly in the number *d* of automata but is nearly independent of the block size. This indicates that the number of iteration steps is independent of changes in the ordering of automata and consequently of changes in the rank.

## Conclusion and future work

Inspired by current research in tumor progression models, we considered a class of continuous-time Markov chains that describe interacting processes, e.g., tumor progression models. Typically the age of a tumor at its diagnosis is unknown. For this reason, the transient distribution integrated over the exponentially distributed observation time is required. This so-called time-marginal distribution is uniquely defined as the solution a large linear system and suffers from the problem of state-space explosion. We modeled this class of Markov chains with separable transition rates factorizing according to the current state using Stochastic Automata Networks. This modeling enabled us to obtain a low-rank tensor representation of the operator and the right-hand side of this linear system. Based on these low-rank tensor representations, we derived an iterative method to compute a low-rank tensor approximation of the time-marginal distribution and hence were able to overcome the state-space explosion. The method guarantees that the entries of the approximation sum up to one as required for a probability distribution. We proved the convergence of the method. In numerical experiments focused on the concept of Mutual Hazard Networks we illustrated that the time-marginal distribution is well approximated with low rank. The method allows for consistently low ranks during the iteration, and linear convergence was observed independently of the number of processes/automata.

A probability distribution, in addition to being normalized to one, must be non-negative. An approximation of a probability distribution should also satisfy this condition. In numerical experiments we observed that our method preserves this non-negativity for small numbers of automata if the truncation is sufficiently accurate. How to guarantee non-negativity and at the same time convergence for high numbers of automata will be part of our future research. Moreover, we observed that the approximation rank for the time-marginal distribution depends strongly on the structure of the dimension tree and on the effects between automata. To minimize the approximation rank we plan to develop a strategy to determine an optimal dimension tree a-priori.

## Data Availability

For the corresponding data and code, please contact the authors.

## Footnotes

1 Note that in other areas it is also common to restrict $\mathsf {Q}$ to have *row* sum 0. Here we use column sum 0 consistently with tumor progression literature. All results can be easily transferred by transposing.

2 For brevity, we do not introduce the concept of functional transitions and restrict ourselves to constant ones, since the separability in (5) can be exploited for reformulation as constant transitions. We refer the interested reader to (Plateau and Stewart 2000) for further details.

3 Strictly speaking, the computational cost of the method is therefore quadratic, rather than linear, in *d*. However, $\gamma$ only needs to be precomputed once, the computational cost of which is negligible.
